# Supplementary material for: Implementation fidelity in leprosy care and support for disability prevention and management in Rupandehi, Nepal: A qualitative study
Source: PLoS One. 2025 Jul 9;20(7):e0327465. doi: 10.1371/journal.pone.0327465 (PMC12240290; doi:10.1371/journal.pone.0327465)
Supplement: S1 Table — (DOCX) [file pone.0327465.s001.docx]

**Table:4 codebook**

| **S.N** | **Themes** | **Sub-themes** | **Codes** |
| --- | --- | --- | --- |
| 1 | Healthcare provider’s adherence to leprosy services | Case and complication management for disability prevention | Treatment protocols and guidelines, cardinal signs, categorization of leprosy, referral to tertiary hospitals, confirmation examinations, multi-drug therapy, recordkeeping, complications, complication management. |
|  |  | Adherence to leprosy operational guidelines | Knowledge and awareness of treatment protocols and guidelines, access to treatment protocols and guidelines documents, follow treatment protocols and guidelines, accurate treatment services, involvement of family members and FCHVs. |
|  |  | Follow up and monitor case registration through MDT | Client follow-up process, voluntary muscle testing, sensory testing, skin smear test, referral service. |
| 2. | Healthcare provider's experience towards quality leprosy services | Accessibility of the service | Un/availability of skin smear tests, skills of lab personnel, qualification of lab personnel, un/availability of essential diagnostic kits, access to referral service, types of leprosy services provided by health institutions, satellite clinics, tertiary-level health facilities. |
|  |  | Timeliness of services | Prompt action on suspected cases, start of the assessment procedure, timely confirmation of the leprosy status, timely referral to referral sites, adequate human resources at service sites, and adequate budgeting for health sites. |
|  |  | Skills of health care providers | Need for specific complication management training, need for refresher training, onsite coaching, and peer education. |
| 3. | Participants' responsiveness to the leprosy services | Satisfaction towards services among people with leprosy | Adverse effects of medicines, pills burden, free basic leprosy services, insufficient resources for complication management, assistive devices |
|  |  | Level of engagement in self-care | Counselling services, member of self-help group (SHG) and self-care practice training |
|  |  | Perceived stigma in accessing leprosy services | Social stigma, social discrimination, privacy of the leprosy condition, isolation from the family, isolation from society, fear of discrimination, loss of jobs due to discrimination. |
|  |  | Barriers and facilitators to leprosy services | Financial viability, expensive treatment of complicated cases, institutional and systemic barriers, effective communications including privacy and confidentiality, role of external development partners and provision of transportation incentives, support from local government, high out-of-pocket expenditures during referrals, superstitious beliefs, difficult to travel, inadequate resources, lack of skilled human resources, unresponsiveness of healthcare providers, inconsistent drug supply, involvement of family members, involvement of female community health volunteers (FCHVs), effective communications, maintenance of privacy and confidentiality for people with leprosy, provision of transportation incentives. |
| 4 | Strategies to facilitate implementation fidelity | Support from key stakeholders | free medicine, provision of transportation incentives, reward system. |
|  |  | Social Security Provisions and mechanism |  |
|  |  | Extending outreach services in endemic areas |  |
