# Supplementary material for: Implementation fidelity in leprosy care and support for disability prevention and management in Rupandehi, Nepal: A qualitative study
Source: PLoS One. 2025 Jul 9;20(7):e0327465. doi: 10.1371/journal.pone.0327465 (PMC12240290; doi:10.1371/journal.pone.0327465)
Supplement: S2 File — (DOCX) [file pone.0327465.s002.docx]

## **Informed consent**

**I. Request for Participation**

I am Sudip Nepal, conducting research titled “**Implementation fidelity in leprosy care and support for disability prevention and management in Rupandehi, Nepal: A qualitative study”** for my study at Universitas Gadjah Mada, Indonesia. I would like to have a discussion with you about your experiences towards care and support of quality leprosy services for disability prevention and management and participants' responsiveness to the care and support of leprosy services for disability prevention and management through their reported experiences and perceptions. Your participation is vital for the fulfilment of my study, and in helping understand the adherence of healthcare providers to the care and support of people with leprosy for disability prevention and possible barriers and facilitators.

**Risk of Research:** The participants attending this research have no physical or mental risk. The investigator requests you to provide your time to answer a few questions in an interview. Some information may also be taken during observation. It will take just 30-60 minutes.

**Privacy secured:** We will do our best to protect the information we collect from the participants. Information which identifies the participants will be kept secure and restricted. This information will be used only for research purposes and the information that you provide will be used solely for study. Apart from some anonymous information, your name will not be written on the interview note or anywhere else and will never be used in any part of the research. Participation in this survey is voluntary and you may choose not to answer some questions if you find them not necessary and withdraw and end the interview at any time.

**Participation benefits:** Your contribution to this study will be for research purposes only. This research outcome can contribute to the care and support of people with leprosy for disability prevention and management in Rupandehi, Nepal. I therefore would greatly appreciate your help in responding to the interview. I am ready to clarify for you if you have any doubts regarding the process or any issues you may feel before the discussion. You are being requested to participate in this research project and to give your consent. Before participating, if you don’t realize anything, ask the investigator. If you agree to participate in this study, you will be asked to sign this consent form.

**II. Consent Form**

I hereby give my consent to participate in the study titled ***Implementation fidelity in leprosy care and support for disability prevention and management in Rupandehi, Nepal: A qualitative study* Researcher**: Mr. Sudip Nepal, Student at the Universitas Gadjah Mada, Indonesia

I fully understood and agreed that the purpose of the interview and discussion is solely for research purposes. I have thus agreed to participate if my identity will be treated with confidentiality and my name will not be used anywhere in the research. I have consented to participate in the study even if it is published and it does not reveal my name and identity whatsoever concerning the research findings. I have also been informed that I will not be forced to give any information I don’t want and that I can withdraw from participation in the interview and discussion at any time without giving reasons for doing so.

Signature ……………………………………...Date ………………………………………..

You may have queries now or later. For questions about the study, please contact principal investigator Sudip Nepal at nepalsudip25@gmail.com you can call +9779847586512. You can also contact two of the advisors of this research project dr. Riris Andono Ahmad at [risandono_ahmad@ugm.ac.id](mailto:risandono_ahmad@ugm.ac.id) or Prof. dr. Ari Probandari at [ari.probandari@gmail.com](mailto:ari.probandari@gmail.com). You can also ask questions about the research to the Medical and Health Research Ethics Committee of the Faculty of Medicine UGM (Tel. 0274-588688 ext 17225 or + 62811-2666- 869; email: [mhrec_fmugm@ugm.ac.id](mailto:mhrec_fmugm@ugm.ac.id))

Thank you very much.
