# Supplementary material for: Implementation fidelity in leprosy care and support for disability prevention and management in Rupandehi, Nepal: A qualitative study
Source: PLoS One. 2025 Jul 9;20(7):e0327465. doi: 10.1371/journal.pone.0327465 (PMC12240290; doi:10.1371/journal.pone.0327465)
Supplement: S3 File — (DOCX) [file pone.0327465.s003.docx]

### **A. Tools to observe satellite clinic (Researcher observation checklist)**

**General Information:**

Date of Observation: Observer Name:

Satellite Clinic Name: Location:

1. Are people with leprosy experiencing delays in receiving their treatment or services? Explain

2. Has health care provider taken comprehensive leprosy training for the care and support of people with leprosy for disability prevention and management?

3. Are there any bottlenecks or challenges while providing care and support to leprosy services? Like language and communication barriers explain

4. Healthcare provider's knowledge about leprosy symptoms, diagnosis, and complication management? Explain

5. How is the referral mechanism for managing leprosy-related complications? Please explain

6. Can you elaborate on the types and range of services the healthcare provider offers to people with leprosy for complication management?

7. Is there any practice to monitor the people with leprosy's progress through multi-drug therapy (MDT)? Explain

8. Please explain the healthcare provider's engagement with people with leprosy in self-care.

9. Please explain the barriers to service in satellite clinics for the care and support of people with leprosy for disability prevention and management.

10. Please explain the facilitators of service in the satellite clinic for the care and support of people with leprosy for disability prevention and management.

11. Are people with leprosy satisfied in terms of care and support services provided for disability prevention and management? Explain

**Overall Assessment:**

How is the overall fidelity of leprosy services at the satellite clinic?

### **Tools to observe health facilities (Researcher observation checklist)**

**General Information:**

Date of Observation: Observer Name:

Health Facility Name: Location:

1. Are people with leprosy experiencing delays in receiving their treatment or services? Explain

2. Has health care provider taken comprehensive leprosy training for the care and support of people with leprosy for disability prevention and management?

3. Are there any bottlenecks or challenges while providing care and support to leprosy services? Like language and communication barriers explain

4. Healthcare provider's knowledge about leprosy symptoms, diagnosis, and complication management? Explain

5. How is the referral mechanism for managing leprosy-related complications? Please explain

6. Can you elaborate on the types and range of services the healthcare provider offers to people with leprosy for complication management?

7. Is there any practice to monitor the people with leprosy's progress through multi-drug therapy (MDT)? Explain

8. Please explain the healthcare provider's engagement with people with leprosy in self-care.

9. Please explain the barriers to service in health facilities for the care and support of people with leprosy for disability prevention and management.

10. Please explain the facilitators of service in health facilities for the care and support of people with leprosy for disability prevention and management.

11. Are people with leprosy satisfied in terms of care and support services provided for disability prevention and management? Explain

**Overall Assessment:**

How is the overall fidelity of leprosy services at the health facilities?

1. **Tools for Key informant interviews (KIIs) of healthcare provider**

**Participants Demographics:**

Name: Professional role: Age:

Health Facility Name: Gender: Years of experience:

**1.** Please explain the range of care and support services that healthcare provider offers to people with leprosy for disability prevention and management.

2. Do you monitor the people with leprosy progress through MDT? How often follow up is made and what’s the follow-up process?

**Satellite clinic:**

3. Do you refer people with leprosy to a monthly satellite clinic at Butwal? How beneficial do you think monthly satellite clinics are for both people with leprosy and health care providers in terms of care and support for complication management and disability prevention?

4. Have you ever received Comprehensive Leprosy Training (CLT) provided to healthcare providers to enhance their knowledge and skills in leprosy care?

***If yes, please share your experience and how it is helping you to care for and support leprosy cases for disability prevention.***

5. How familiar are you with Nepal’s leprosy operational guidelines/ protocols?

Are you providing services as per guidelines/protocols? Why/why not? Any difficulties?

6. Is care and support for people with leprosy easily available for disability prevention and management?

***How do you ensure prompt service for people with leprosy for disability prevention and management?***

7. How do you ensure correct diagnosis for leprosy cases in your facility?

***Are there any facilitators or barriers to achieving correct diagnoses?***

8. How do you provide care and support to complicated leprosy cases such as ulcer, reaction, and disability cases?

***What are the major complaints or feedback of clients regarding services?***

9. Do you refer the client? Please explain about referral mechanism for complicated cases like ulcers, reactions, and pain management.

***Where do you refer those patients that are not managed in health facilities and processes?***

10. What are the barriers to providing care and support to people with leprosy for disability prevention and management? If yes, please elaborate.

11. What are the facilitators of providing care and support to people with leprosy for disability prevention and management? If yes, please elaborate.

### **Focussed Group Discussion (FGDs) Tool for SHG Members**

**Qualitative Research Tools FGD**

Name of SHG: Total Participants:

Date of FGD conducted:

1. Can you share your experiences regarding the availability of leprosy services in health facilities for complication management like ulcer care, and reaction management?
2. Could you please explain the accessibility of leprosy service in terms of cost?
3. Are you satisfied with the quality of services provided to people with leprosy?

( Is treatment benefitting you? What about pill burden?)

1. How can the quality of leprosy services be ensured? What kind of services do you prefer for complication management and disability prevention?

5. How do you take care of yourself regarding leprosy treatment?

(Probe: Number of dose intake, timely intake of medicine, self-care)

1. How engaged is your healthcare provider in teaching self-care practices to you? Would it be better if your healthcare provider taught you about self-care?
2. How do you perceive stigma to access leprosy services for complication management? What specific challenges do you encounter in seeking and receiving appropriate care and support for leprosy services?

8. Please explain about counselling and emotional support provided by health care providers that made it easier for you to access leprosy services for disability prevention and management. Please elaborate.

9. Have you experienced assistive devices like MCR footwear, wheelchairs, prosthetic devices and other devices that made it easier for you?

10. Have you encountered any facilitators that made it easier for you to access leprosy services for disability prevention and management? Please elaborate.

11. Have you encountered any language and communication barriers from health care providers due to poor communication skills that made it difficult to access leprosy services for disability prevention and management? Please elaborate.

12. Have you encountered barriers from health care providers due to limited specialized care services for disability prevention and management? Please elaborate.

1. Have you encountered any barriers that made it difficult for you to access leprosy services for disability prevention and management? Please elaborate.
