# Supplementary material for: Implementation fidelity in leprosy care and support for disability prevention and management in Rupandehi, Nepal: A qualitative study
Source: PLoS One. 2025 Jul 9;20(7):e0327465. doi: 10.1371/journal.pone.0327465 (PMC12240290; doi:10.1371/journal.pone.0327465)
Supplement: Attachment — Submitted filename: Author_Response_Leprosy_Implementation Fidelity.docx.docx [file pone.0327465.s005.docx]

Editor-in-Chief
PLOS ONE

**Subject: Revisions for Manuscript [PONE-D-25-09075] – "Implementation fidelity in leprosy care and support for disability prevention and management in Rupandehi, Nepal: A qualitative study"**

Dear Editor,

We sincerely appreciate the time and effort you and the reviewers have invested in evaluating our manuscript. This detailed feedback has been invaluable in helping us to refine and strengthen our work. We have carefully considered all comments and revised our manuscript accordingly. Below, we provide a detailed response to each point raised by the reviewers. All changes have been made in the revised manuscript for easy reference.

Below, we provide a point-by-point response to each comment. We have also uploaded a tracked-changes version and a clean version of the revised manuscript for your review.

We sincerely appreciate your guidance throughout this process. Please feel free to reach out if you have any additional questions or concerns.

On behalf of co-authors,

Sudip Nepal

[nepalsudip25@gmail.com](mailto:nepalsudip25@gmail.com)

| **Editor’s/Reviewer’s comments** | **Response** | **Corrected page#, and lines#** |  |
| --- | --- | --- | --- |
| **Editor’s Comment** | | | |
| 1.Please ensure that your manuscript meets PLOS ONE's style requirements, including those for file naming.  https://journals.plos.org/plosone/s/file?id=wjVg/PLOSOne_formatting_sample_main_body.pdf and  https://journals.plos.org/plosone/s/file?id=ba62/PLOSOne_formatting_sample_title_authors_affiliations.pdf | Thank you. We have ensured that our manuscript meets PLOS ONE's style requirements. We have addressed in the main document and all those supporting files accordingly. |  |  |
| 2**.** Please include a complete copy of PLOS’ questionnaire on inclusivity in global research in your revised manuscript. Our policy for research in this area aims to improve transparency in the reporting of research performed outside of researchers’ own country or community. The policy applies to researchers who have travelled to a different country to conduct research, research with Indigenous populations or their lands, and research on cultural artefacts. The questionnaire can also be requested at the journal’s discretion for any other submissions, even if these conditions are not met.  Please find more information on the policy and a link to download a blank copy of the questionnaire here: <https://journals.plos.org/plosone/s/best-practices-in-research-reporting>. Please upload a completed version of your questionnaire as Supporting Information when you resubmit your manuscript. | A complete copy of the PLOS questionnaire on inclusivity in global research has been attached as supporting information. |  |  |
| 3. In the ethics statement in the Methods, you have specified that verbal consent was obtained. Please provide additional details regarding how this consent was documented and witnessed, and state whether this was approved by the IRB | The study received an ethical approval letter from the Ethical Board Committee, Universitas Gadjah Mada, (UGM) Indonesia, (Reference no: KE/FK/0291), Nepal Health Research Council (NHRC),(Reference no: 1078), and Health Office Rupandehi (Reference no: 208). Ethical approval covered all study procedures, including the consent process. Verbal and written informed consent was obtained from all participants. The consent form was approved by both ethical committees from Nepal and Indonesia, and participants were informed about the study's purpose, confidentiality, voluntary participation, and the right to withdraw at any time. Verbal consent was documented by the research team in the form of audio recordings and written consent was obtained from participants before the recordings were taken and signed by the interviewer, as per the approved ethical protocol. | # Page 17 and 18  From lines 292 to 299. |  |
| 4. We note that the grant information you provided in the ‘Funding Information’ and ‘Financial Disclosure’ sections do not match.  When you resubmit, please ensure that you provide the correct grant numbers for the awards you received for your study in the ‘Funding Information’ section. | The primary author, Sudip Nepal, received a scholarship from the World Health Organization Tropical Disease Research (WHO-TDR) Special Postgraduate Programme in Implementation Research at Universitas Gadjah Mada (UGM), Indonesia, to pursue a Master's in Public Health(MPH), and this study was carried out as part of his thesis. So there is no specific grant number available for this thesis study. This is revised and updated in the funding information section. |  |  |
| 5. When completing the data availability statement of the submission form, you indicated that you will make your data available on acceptance. We strongly recommend all authors decide on a data sharing plan before acceptance, as the process can be lengthy and hold up publication timelines. Please note that, though access restrictions are acceptable now, your entire data will need to be made freely accessible if your manuscript is accepted for publication. This policy applies to all data except where public deposition would breach compliance with the protocol approved by your research ethics board. If you are unable to adhere to our open data policy, please kindly revise your statement to explain your reasoning and we will seek the editor's input on an exemption. Please be assured that, once you have provided your new statement, the assessment of your exemption will not hold up the peer review process. | The data from this study are fully available within the paper and its supporting information file for open access during the initial submission. The data availability statement is corrected accordingly in the submission form. |  |  |
| 6. Please include captions for your Supporting Information files at the end of your manuscript, and update any in-text citations to match accordingly. Please see our Supporting Information guidelines for more information: [http://journals.plos.org/plosone/s/supporting-information](http://track.editorialmanager.com/CL0/http:%2F%2Fjournals.plos.org%2Fplosone%2Fs%2Fsupporting-information/1/010f0195c709ff44-b1393dd6-545c-4fe6-9ae3-c2ea7f4183ed-000000/6MKxEV127JzeB-dU4tm8qEBITNNacGnifJ5W9P7kORA=203). | The necessary changes have been made, and the supporting information files have been renamed as per the journal's guidelines. |  |  |
| **Additional editors comment** |  |  |  |
| 1. The paper talks about implementation fidelity but does not bother comparing and contrasting with country clinical practice guidelines or leprosy control program operations manual or WHO best practices for leprosy. How are you able to conclude that there was implementation fidelity (or there was none)? Please consider adapting the methodology used here where we compared what the facilities found with what is the recommended practice by our ministry of health and the World Health Organization: <https://actamedicaphilippina.upm.edu.ph/index.php/acta/article/view/409> | Thank you for your insightful feedback. We recognize the importance of systematically comparing observed practices with both national and international guidelines. So, we have systematically compared our findings with standard [**National Leprosy Operational Guidelines (2075).**](../../../../Downloads/National%20leprosy%20operational%20guideline.pdf) **Nepal's leprosy operational guidelines have been adopted and are aligned with** [**WHO guidelines.**](../Desktop/leprosy%20protocol_Nepal.pdf) The comparison has been presented in Supplementary Tables (S1 and S2), where we analysed healthcare provider adherence across key dimensions such as case management, adherence to leprosy protocol, follow-up, and self-care involvement. Any observed deviations from the recommended practices have been discussed and how the observed fidelity aligns or deviates from the national leprosy operational guidelines ensures the implementation fidelity. |  |  |
| 2. What is the theoretical framework used in analyzing the study? How were the themes generated and what is the basis for presenting these themes? | Thank you for your question. The study utilized the **Carroll et al. (2007) framework** to explore the implementation fidelity of leprosy care and support for disability prevention and management. The framework consists of several domains, including: Adherence, dose, quality of delivery, participant responsiveness, strategies to facilitate and program differentiation.  In our study, we have explored the quality of delivery, participant responsiveness, strategies to facilitate and adherence.  When we generated the themes, we adapted the domains in the Carroll et al.' framework and confirmed the study objectives and themes were generated based on study objectives. For example: under the theme of Healthcare provider’s experience to quality leprosy services, there are sub-themes of explored accuracy, timeliness, and accessibility of services **(linked to quality of delivery) as per the Carroll et al framework of fidelity.**  Under the theme of Participant Responsiveness, there are sub-themes of self-care practices, Satisfaction towards services and stigma, Barriers and Facilitators: Identified institutional, financial, and social factors. **(linked to participant responsiveness) as per the Carroll et al framework of fidelity.**  Healthcare provider adherence to leprosy services: Assessed protocol compliance, case and complication management **(linked to adherence) as per the Carroll et al framework of fidelity.** |  |  |
| 3. What is the reflexivity and positionality of the authors as they did this study? Please add this to the manuscript. | “The researcher consistently reflected throughout the research process, critically examining their biases and assumptions. By maintaining awareness of these influences, they aimed to minimize bias and enhance the credibility of their findings. Furthermore, the researchers approached the study with cultural sensitivity, recognizing that their positionality could influence how data is interpreted and analysed. Therefore, they strived for objectivity while acknowledging how their backgrounds might shape their interpretations. Multiple data sources and interviewing diverse participants ensured the credibility and dependability of the findings”  This information is updated in the methodology section of the manuscript accordingly. | #page 17  From lines 280 to 287 |  |
| 4. While you are discussing adherence to protocols, can you also discuss treatment extension, the fidelity of healthcare providers in keeping with treatment guidelines, and its effect on patient adherence to medication? <https://www.cell.com/heliyon/fulltext/S2405-8440(21)01382-7> | Thank you so much for the constructive feedback. We have now expanded the discussion to address the topics of treatment extension, healthcare providers’ fidelity to national treatment guidelines, and their collective influence on patient adherence to medication in manuscript.  “The use referral slips from health facilities with accurate diagnoses and contact details made the referral process simpler to receive specialized care and support. Furthermore, the engagement of FCHVs in the treatment process was found to have assisted in treatment adherence by suspecting and referring the cases, and following up on leprosy cases.”  “Healthcare providers engaged in complication management through quarterly VMT/ST, basic pain management, self-care training and timely referral of complicated cases to Anandaban Hospital.”  “The majority of healthcare providers showed extensive knowledge of cardinal signs and MDT regimens which demonstrate sufficient adherence to national leprosy operational guidelines. This is foundational knowledge for healthcare providers for an accurate diagnosis”  “Our findings suggest healthcare providers maintained standardized care across several healthcare facilities due to national leprosy operational guidelines clarity. In addition, adherence to MDT regimens for specified durations (6, or 12 months) ensured appropriate and effective treatment. In certain cases, particularly those with persistent complications treatment durations were extended based on clinical judgement. These treatment extensions were implemented following national leprosy operational/WHO guidelines, and healthcare providers' adherence to treatment extensions helped to improve patient outcomes.”  “The follow-up strategies such as regular contact through phone calls, home visits, and involvement of FCHVs and treatment supporters ensured continuous care, compliance and treatment adherence” | # page no 33 to 35  From lines 565 to 600. |  |
| 5. Discussing self-care for leprosy is good, but I think there is a need to be cautious about what self-care practices are good and what self-care practices are bad: <https://academic.oup.com/heapol/article/38/2/205/6798855?login=false> <https://www.emerald.com/insight/content/doi/10.1108/ijhg-01-2023-0008/full/html> | Thank you for constructive feedback. Good self-care and bad self-care have been incorporated in the introduction paragraph.  “ Individuals may adopt harmful practices such as using inappropriate oils or herbal remedies, excessive soaking of wounds, or delayed wound care. Good self-care practices like routine wound cleaning, protective footwear, and regular self-inspection can all significantly lower the risk of ulceration and disability. | #Page 7  From lines 141 to 146 |  |
| 6. While it was done in passing, can you please give a more detailed description of Nepal's healthcare system and the provision of leprosy services to familiarize the international reader about the paper? Specifically, please mention how long is the travel time from you to referral hospitals and other context to fully appreciate the issues that you have discussed, or if you are from an urban area or a rural area? | Thank you for the comment. We have now expanded the introduction section to incorporate and address a detailed description of Nepal's healthcare system, the provision of leprosy services, the travel time from referral hospitals, and other contexts to fully appreciate the issues and familiarise the international reader.  “ The healthcare system in Nepal can be categorised into three levels: primary (health posts), secondary (district hospitals), and tertiary (specialised institutions). Leprosy services are decentralised, with health posts serving as the primary point of contact for individuals seeking healthcare in the community. This initial contact serves as the first point for leprosy services such as leprosy diagnosis, MDT initiation, disability grading, complications management, referral and follow-up. The key health care providers responsible for implementing leprosy care and support services in the health post include the health post-in-charge and the leprosy focal person who has either a health assistant or auxiliary health worker background with leprosy training. To guide the healthcare providers, Nepal has standard leprosy operational guidelines adopted and aligned with WHO guidelines to facilitate the implementation of care and support of leprosy for disability prevention and management in health facilities such as health posts, hospitals, and referral centres”  “Despite these efforts, health posts in Rupandehi district lack essential skin smear tests, and skilled laboratory personnel to perform these tests. Consequently, they rely heavily on referrals. Participants from remote areas of Rupandehi district must travel 1-2 hours to reach Butwal for a skin smear test. Thus, healthcare providers refer patients to Butwal for these tests and to specialised hospitals in Kathmandu for leprosy-related complications, such as lepra reaction and ulcer management, if local health facilities cannot address them. Participants from these remote regions face considerable challenges due to transportation difficulties, often needing to travel 10-12 hours to reach specialised hospitals like Anandaban in Kathmandu for managing leprosy complications, resulting in out-of-pocket expenses, long waiting times and inconvenience for socio-economic disadvantaged populations” | # Pages 6 and 7  From lines 114 to 137. |  |
| **Reviewer #1** |  |  |  |
| **Background** Your background section emphasizes the importance of implementation fidelity but lacks a clear rationale for studying it in the context of leprosy care in Nepal. To strengthen the justification, briefly explain why exploring implementation fidelity is crucial for improving leprosy care in Nepal. Also, a shorthand definition to implantation fidelity, could be beneficial to the readers unfamiliar to the topic. | Thank you very much for the feedback. We have expanded the introduction section to incorporate the implementation fidelity definition and rationale for studying in the context of leprosy and improving leprosy care in Nepal.  “ Implementation fidelity refers to the degree to which an intervention or program is delivered as intended by the program developers. High fidelity ensures that established leprosy recommendations and protocols are properly implemented by healthcare providers, resulting in better patient outcomes. In contrast, low fidelity explains why these underperform while being well-designed. Assessing implementation fidelity allows researchers to better understand how and why an intervention works, as well as the extent to which outcomes might be improved”  “ The relevance of studying implementation fidelity in leprosy care and support in the Rupandehi district lies in understanding how effectively Nepal’s leprosy operational guidelines are implemented at the local level. Although national prevalence may be low, persistent leprosy complications, G2D cases, and stigma in leprosy-endemic districts like Rupandehi indicate gaps in policy and practice, justifying this research. While grey literature briefly discusses implementation fidelity in leprosy care and support, peer-reviewed studies systematically exploring fidelity in low-resource settings like Nepal remain scarce. This gap limits the development of evidence-based strategies aimed at enhancing service delivery”  “This study explores the implementation fidelity of leprosy care and support for disability prevention and management in the leprosy-endemic region of Rupandehi, Nepal which ultimately bridges the gap between existing leprosy policies and their practical application. Furthermore, this study provides empirical evidence, enabling healthcare providers and policymakers to understand the barriers and facilitators affecting implementation fidelity and suggest tailored interventions to enhance the quality of service delivery” | # Page 7 and 8  From lines 150-164 |  |
| **Methodology** Your methodology section effectively outlines a numerous qualitative methods used, but specifying the number of participants would enhance clarity. Also, if applicable, please rearrange your sentences in the sequences the event takes place. For example, if sampling design is conducted before data collection. Also, mention the timeline the study was conducted.  Although ‘thematic data analysis’ is a used frequently, I think the correct term for qualitative research method is ‘thematic analysis.’ | Thank you for the feedback. We have expanded the methodology section in main manuscript to address the participants number, timeline of the study and re arranging the sentence in sequence throughout the main manuscript accordingly.  **Study design:**  “A qualitative case study design was adopted, grounded in the principles of implementation research. Data were collected through multiple sources and methods like key informant interviews (KIIs) with healthcare providers (n=12), focus group discussions (FGDs) with self-help group members (SHGs) (n=14), and qualitative observation at two high-caseload health facilities and a satellite clinic (n=3) to ensure triangulation and enhance the credibility of the findings.”  **Participant selection**  “Participants were selected via purposive and selective sampling, ensuring alignment with leprosy service availability and geographical locations. This allowed for a rich and in-depth exploration of the research question until data saturation. The data collection period for the study was from 25^th^ February to 30^th^ April 2024, with participant enrolment beginning on 25^th^ February 2024.”  Thematic data analysis has been corrected with thematic analysis throughout the manuscript. | # Page 8-18  From lines 184-190  From lines 192-196 |  |
| **Results & Conclusions**  As per author guidelines, you are discouraged to use abbreviation in the abstract. Please revise accordingly.  After reading your results and conclusions section in the abstract, I would like to suggest that you should consider revising the title of your study. Is your study specific to the role of healthcare providers in leprosy prevention and management or is it about overall implementation fidelity in leprosy care?  Please ensure the consistency of ‘tense’ in your sentences. | Thank you so much for the suggestion abbreviation in the abstract has been removed and revised accordingly.  Thank you for your thoughtful comment regarding the title of the manuscript. We understand your concern and appreciate the opportunity to clarify. While healthcare providers play a significant role in our findings—as they are key implementers of leprosy services—the primary focus of this study remains on the **overall implementation fidelity** of leprosy care and support for disability prevention and management, in alignment with the national leprosy operational guidelines. We have carefully reviewed the title and believe it appropriately reflects the broader scope of our research. Changing the title to focus solely on healthcare providers might narrow the perceived scope and create confusion about the intent and objectives of the study.  To address your concern, we have slightly revised the **abstract results and conclusion** sections to clarify that healthcare providers are part of the broader system under examination, and not the exclusive focus. |  |  |
| **Overall** Please refer to the PLOS ONE author guidelines. Your abstract is at least 23 words above the permitted word limit. Your abstract is comparatively well-written in the introduction and methods section, while you may consider tightening your sentences in the results and conclusions sections, particularly focusing to reduce redundancy. Otherwise, this is a good abstract. | The abstract has been restructured. Redundancy has been reduced, and a new abstract word limit is 270 words including keywords. | # Page 2, and 3 |  |
| **Introduction**  First paragraph  Please cite a source that listed 23 prioritized countries on leprosy. As Nepal does not locate under Southeast Asia, replacing the regional prevalence of Southeast Asia to South Asia would be helpful. If this dataset is not available, you may cite the prevalence in neighboring and some South Asian countries. The sociocultural context of Southeast Asia may not closely resemble that in South Asia. Also, please quantify to what extent the cases of leprosy was reduced during the COVID-19 pandemic. Then, what’s the post-pandemic status of leprosy reporting? | Thank you so much for the feedback. The introduction is revised as per feedback.  The source for 23 prioritized countries on leprosy has been cited in the main manuscript.  “Leprosy remains a public health concern, especially in 23 globally prioritised countries, including Nepal. As per data from 2023, In South Asia, India continues to report more than 10,000 new cases, while other countries (Bangladesh, Nepal and Sri Lanka) each reported 1000–10,000 new cases. Although Nepal is geographically located in South Asia, early references often included it in Southeast Asia's statistics due to WHO regional classification with Nepal contributing to 2% of the total burden in South Asia. As per WHO reports, globally, new cases decreased by 19.3% between 2013 and 2022. The COVID-19 pandemic reduced the detection of new cases due to restricted health service access and reduced active case finding. For example, new case detection in Nepal dropped markedly in 2020 with the reporting of 2173 new cases but began to rebound in the post-pandemic period, with the country reporting 2,523 new cases in the fiscal year 2022/23” | #Page 4 to 8  #Page 4  lines 83 to 93  #Page 4 and 5  Lines 70 to 76  Lines 91-93  # Page 4  Lines 79-81  #Page 5  Lines 101-112  #Page 5  Lines 95-99  #Page 6  Lines 114 to 126  #Page 8  Lines 171-173 |  |
| Second paragraph  Please begin with presenting leprosy as a significant health problem in the history of Nepal, and then what the current status is. While you present the facts and figures, please make it comprehensible for the readers.  For example, first clarify if leprosy is a disability? Disability and disease are different constructs. Then, explain the meaning of MDT. How severe are cases of leprosy of Grade 2 Disability and how is it different than other Grades?  It is evident that since 2010 leprosy was not a significant health problem at national level. However, how was/is the burden of leprosy in Rupandehi, Nepal? If this is not at a significant level, what is the significance of this study? Or, was/is the prevalence of leprosy in the study area underestimated? If yes, for what factors? | Leprosy as a significant health problem in the history of Nepal and its current status has been incorporated in the main manuscript.  “Leprosy, also known as Hansen’s disease, has historically been one of the most stigmatised and significant public health issues in Nepal. The national leprosy elimination program has been implemented in Nepal since 1913/14, with ongoing government efforts to support people with leprosy. Though Nepal achieved the milestone of eliminating leprosy as a public health problem in 2010, defined by the World Health Organization (WHO) as a registered prevalence of less than 1 case per 10,000 population. However, challenges such as early case diagnosis, complication management and disability prevention persist”  “New case detection in Nepal dropped markedly in 2020 with the reporting of 2173 new cases but began to rebound in the post-pandemic period, with the country reporting 2,523 new cases in the fiscal year 2022/23”  Disability and disease construct has been explained in the main manuscript.  “It is essential to note that leprosy is a disease, whereas disability is a potential consequence that may result from untreated or inadequately managed leprosy”  MDT information and Grade 2 disability-related information have been explained in the main manuscript.  “ MDT is the WHO-recommended combination of antibiotics consisting of rifampicin, dapsone, and clofazimine administered over 6 to 12 months depending on the leprosy categorization to eliminate infection and prevent resistance. The WHO classifies leprosy according to the WHO disability grading system, where grade 0 means no impairment, grade 1 means loss of sensation in the hand, eyes or foot, and grade 2 indicates a higher degree of disability compared to grade 1, G2D indicates visible deformities or damage in eyes, hands, or feet at diagnosis indicating more significant functional impairment and potential for long-term complications”  The burden of leprosy in Rupandehi and related explanation has been done in the main manuscript.  “Rupandehi district has historically been one of the leprosy-endemic districts in Nepal. **It still carries a notable leprosy burden,** possibly **underestimated due to multiple socio-health system factors.** Rupandehi district has a registered prevalence of 1.2 per 10,000 population as of 2024. New case detection rates in Rupandehi have often been **above the national average** and grade 2 disability(**G2D) cases** still occur, indicating **missed or late detections**.” |  |  |
| Third paragraph  Explain what health post in Nepal is. Is it the lowest level of healthcare access to Nepali populations?  In the second paragraph, it is better for readers to explicitly specify how many times the data was collected or what months the data was collected. The current version needs to be clarified regarding when the enrolment started. | Thank you for the query. It has been addressed in the main manuscript.  “ The healthcare system in Nepal can be categorised into three levels: primary (health posts), secondary (district hospitals), and tertiary (specialised institutions). Leprosy services are decentralised, with health posts serving as the primary point of contact for individuals seeking healthcare in the community. This initial contact serves as the first point for leprosy services such as leprosy diagnosis, MDT initiation, disability grading, complications management, referral and follow-up. The key health care providers responsible for implementing leprosy care and support services in the health post include the health post-in-charge and the leprosy focal person who has either a health assistant or auxiliary health worker background with leprosy training. The level of health care provider’s care and support required may vary depending on the individual's needs and the stage of the disease. To guide the healthcare providers, Nepal has standard leprosy operational guidelines adopted and aligned with WHO guidelines to facilitate the implementation of care and support of leprosy for disability prevention and management in health facilities such as health posts, hospitals, and referral centres.”  For better flow, data collection times and month details including enrolment date has been explained in the end of final paragraph of the introduction section.  “ In this study, data was collected once over two months, from 25^th^ February to 30^th^ April 2024, with participant enrolment beginning on 25^th^ February 2024.” |  |  |
| Overall  The introduction section has important information but lacks proper flow and some important background information. For example, it lacks regional nation vs region-specific information on leprosy. Adding a context of history of leprosy in Nepal and connecting it to the current status of leprosy can be helpful. Also, socio-cultural environment of Nepal has interplayed an important role on leprosy care and management, which was lacking. To streamline the flow, I suggest you consider revising your introduction as below:  - Present leprosy as a significant public health problem in Nepal’s history, and how why studying leprosy care and management is still important in the current context in spite of its lower prevalence?  - Discuss leprosy care and management from the light of socio-cultural context in Nepal and specific to your study area.  - Discuss how leprosy care is available and what are the major barriers to its prevention and treatment.  - Provide a strong rationale why your study is important given the current scenario of leprosy in the study area. How does your study in a local context be useful to the national level and what are its relevance to the global society? Mentioning that leprosy care and support implementation fidelity is discussed only in grey literature does not provide a strong rationale for your study. Contextualize it more robustly.  - One major limitation in the introduction was the unclear delivery of what the term ‘implementation fidelity’ is. If you think that your study is more about ‘implementation fidelity,’ you will need to clarify this jargon to the readers and centralize your introduction under this term. | Thank you so much for the overall feedback. History of leprosy and current leprosy status, region-specific information, socio-economic context of leprosy, leprosy care and management from the light of the socio-cultural context in Nepal, the barrier to leprosy care, rationale of study given the current scenario of leprosy in the study area, usefulness of study in a local context be useful to the national level and its relevance to the global society, implementation fidelity jargon has been addressed throughout the introduction section and revised introduction section is presented in the main manuscript. | #Page 4-8 |  |
| **Methods**  Ethics statement  Please use proper punctuation. For example, reference no should follow with ‘:’ rather than ‘;’. You don’t need to abbreviate any term you have not used more than three times in your manuscript. Also, explicitly specify which health office in Rupandehi did you seek consent from? Is it the provincial health office, or any ministry or something else? Did you provide a copy of informed consent to the participants? And, did you receive consent before recording the interview? Were there any remuneration or benefits provided to the participants in exchange for their time and contribution to your research? Was proxy permitted? As you have integrated several data collection methods, were the approaches to ethical considerations similar or different to each specific method?  **Study setting**  Provide a citation to justify your statement that Rupandehi is one of the leprosy-endemic districts of Nepal. When you specify the borders, be explicit to differentiate the national versus international borders. It might also be insightful to consider inserting a map depicting the study area. While you have specified 1.2/10,000 prevalence, there are two important pieces of information missing: a) to be considered a rate, you need a time variable; prevalence and prevalence rates are two different ideas in epidemiology; and b) which year is this prevalence of?  Also the following sentences – The government of Nepal (GON) …………milestones of NLEP is not relevant under this subheading.  **Study design and participant selection**  I believe you do not require this subheading.  **Study design**  Correct the case from title to sentence case and be consistent throughout your manuscript.  Please simplify when you explain your study design – qualitative implementation research methodology using a multi-source, multi-method case study design – is very vague. Rather specify the exact approach of qualitative research you implemented, such as case study design, grounded theory, narrative study or something else. Please elaborate the method you used for data collection. | Thank you for the suggestion. The punctuation issue replacing ‘;’ with‘:’ has been fixed.  The term that has not been used more than 3 times in the manuscript is not abbreviated throughout the manuscript.  Regarding consent, it was taken from the district public health office, Rupandehi (old name) but now it is renamed and called as Health Office, Rupandehi.  “Informed consent was provided to all the participants and signed as well. Consent was taken from all the participants before recording an interview.  No financial incentives were provided to participants for their time and contributions to the research. However, all participants were sincerely thanked and appreciated for their valuable insights and time. Proxy participation was not permitted. Only individuals who met the inclusion criteria and provided informed consent were involved in the research. Ethical considerations were consistently maintained across all methods. While ethical principles such as informed consent, confidentiality, voluntary participation, and the right to withdraw at any time were uniformly applied, minor adjustments were made. For instance, during focus group discussions, emphasis was placed on maintaining group confidentiality. In contrast, one-on-one interviews focused more on ensuring privacy and building rapport.”  Thank you so much. The study setting section has been revised.  **Study setting**  “Nepal comprises 77 districts and 7 provinces, divided into three ecological belts: mountain, hilly, and terai belts. Rupandehi district is located in the Terai belt of Lumbini province in southwestern Nepal and covers an area of 1,360 km². It is bordered by Palpa to the north (national border), India to the south (international border), Kapilvastu district to the west (national border), and Nawalparasi district (national border) to the east. Rupandehi is one of the leprosy-endemic districts of Nepal with a registered leprosy prevalence of 1.2 per 10,000 population as of 2024.  **Regarding map:**  Thank you for the suggestion. We agree that a visual representation of the study area would enhance understanding. However, we decided not to include a map due to potential copyright restrictions associated with available maps from government and institutional sources. We have instead provided a detailed textual description of the study area in the Methods section.  The government of Nepal (GON) …………milestones of NLEP have been omitted from the main manuscript.  Study design and participant selection subheadings are omitted from the main manuscript.  **Study design**  The case from title to sentence case has been addressed consistently throughout the manuscript.  Thank you for the constructive feedback. The point you have mentioned has been addressed throughout the main manuscript.  **Study design**  “A qualitative case study design was adopted, grounded in the principles of implementation research. Data were collected through multiple sources and methods like key informant interviews (KIIs) with healthcare providers (n=12), focus group discussions (FGDs) with self-help group members (SHGs) (n=14), and qualitative observation at two high-caseload health facilities and a satellite clinic (n=3) to ensure triangulation and enhance the credibility of the findings” | #Page 17  Line 290-299  #Page 8 and 9  Lines 177-183  #Page 9  Lines 185-190 |  |
| Table 1  Please add a table note specifying the standard used for leprosy categorization. Interestingly, 100% of cases are multi-bacillary. Are all cases of leprosy Multi-Bacillary in Nepal or in the study area? If not, why did you lack variability in your study?  **Study tool and data collection**  Please detail out if your tools were designed in English or Nepali language? How did you confirm the validity of the translation? You have mentioned that the tools were pre-tested but did not detail out how and when? Please add these details and describe what changes were made following the pre-test? Did you utilize any software for transcribing the script?  Data analysis  Please cite the software. Also, explain whether you used Nepali or English (if translated) version for the data analysis. Specify what specific methods were used for focused group vs. KII vs. observation. If all of them were analyzed using same approach, explicitly mention it. | The table note specifying the standard used for leprosy categorisation as per WHO standards has been updated in the manuscript.  “ *Note: The WHO classification system is used to categorise leprosy as either PB or MB. MB cases have more than five skin lesions or positive skin smears, whereas PB cases have five or fewer lesions and negative skin smears.”*  **Clarification Regarding Lack of PB Cases**: While both PB and MB leprosy cases are common in Nepal, all people with leprosy in this study were members of Self-Help Groups recruited for the FGDs, and these two SHGs happened to be comprised only of individuals with MB leprosy. Other SHGs in the research area, including those having PB patients, were not included in the FGD due to purposive sampling. As a result, the lack of heterogeneity in leprosy categorisation in this study does not represent national or district-level distribution, but rather the specific context of the SHGs chosen for this research.  Thank you for the query. All these queries related to study tool and data collection are addressed in the main document.  **Study tool and data collection**  “The tools were initially developed in English and later translated into Nepali. The tools were pre-tested with two healthcare providers and one SHG from similar settings to check clarity and cultural relevance, approximately one week before the actual data collection. Based on pre-testing, minor modifications were made to the flow of questions and wording to ensure clarity during data collection. The local facilitators assisted during an interview to ensure the participant's comprehension. Data were recorded using a digital audio recorder, and principal investigators took field notes. All audio recordings and transcriptions were manually transcribed verbatim by trained independent research assistants and cross-verified with field notes to ensure data quality. The KIIs lasted approximately 30 minutes, whereas FGDs were completed in 60-70 minutes. Participants were encouraged to elaborate on detailed experiences to provide rich information. The data was collected until the data saturation was obtained with the last KII as guided.  We have expanded the data analysis section to address the queries related to Software, translated version for the data analysis, specific methods used for analysis of FGD vs KII vs observation are explicitly mentioned in data analysis section of the main manuscript.  **Data analysis**  “The data analysis followed Braun and Clarke’s thematic analysis framework involving systematic steps to ensure the credible interpretation of the findings and their linkages to the research objectives. First, the research team familiarized themselves with the data by reviewing transcripts and observational notes. The data were transcribed verbatim in Nepali and then translated into English. The English-translated versions were used for coding and thematic analysis. The research assistant and principal investigator validated the data after transcription and ensured the accuracy.  Initial codes were generated through line-by-line coding, capturing key elements such as diagnostic tools and healthcare provider skills. These codes were reviewed and grouped into broader subthemes, such as "Accessibility of the service," which summarized challenges like diagnostic kit availability, referral mechanisms, and human resource gaps. Subthemes were further aggregated to form broader themes and refined to ensure alignment with the data, to support the findings. For instance, participants highlighted delays caused by insufficient diagnostic kits and the financial burden of accessing tertiary care.  To support the analysis, data were coded using qualitative analysis software. We used Deedose software to organize and manage the coded data. Inductive coding allowed for the generation of new codes, as stated in Table 3 and a codebook was developed to ensure consistency among researchers. All types of data (KIIs, FGDs and observation) were analysed using the same thematic analysis with special attention paid to the nature of the data source. A manifest analysis was conducted to analyse the qualitative data and to interpret a comprehensive understanding of facilitators and barriers to the fidelity of leprosy care and support implementation. A preliminary analysis was shared with stakeholders for feedback and data triangulation. These findings were interpreted in the context of national leprosy operational guidelines.” | #Page 12  Lines 219-221  # Page 15 and 16  Lines 240-251  # Page 16 and 17  Lines 254 to 278 |  |
| Results  Adherence to leprosy protocols and guidelines  Interestingly, no detail of what standard protocol (national/international/WHO) used to treat leprosy was provided. Any thoughts?  Also, it was mentioned that the skin smear test was not possible due to the unavailability of a lab technician. Elaborating this in discussion could be insightful. Does this mean that these local health units in Nepal have necessary pre-requisites, but only lack the specific experts?  **Follow-up and monitoring of case registration**  Please integrate some points on how leprosy registration in Nepal is performed and whether or not there is a practice of universal or centralized record for leprosy in Nepal.  The only significant comment I have for results section is it’s a bit lengthy. Try to shorten it. | Thank you for your insightful feedback. We recognize the importance of systematically comparing observed practices with both national and international guidelines. So, we have systematically compared our findings with standard [National Leprosy Operational Guidelines (2075).](../../../../Downloads/National%20leprosy%20operational%20guideline.pdf)  **The national leprosy operational guidelines of Nepal does adopt and align with** [**WHO guidelines.**](../Desktop/leprosy%20protocol_Nepal.pdf)  It is elaborated in the discussion section in the main manuscript.  **“Follow-up and monitoring of case registration:**  Once leprosy cases are confirmed, healthcare provider record and register the case in leprosy treatment register in local health post. The case based details are reported in the demographic health information system 2 (DHIS2), which is national data repository. Although this system ensures a standardized format for case reporting, the practice does not amount to a centralized digital record that is accessible in real-time”.  This information is updated in the manuscript.  The result section is shortened in main manuscript wherever possible to make it concise. | #Page 22  Lines 320 to 322  #Page 23  Lines 343 to 348 |  |
| **Discussion**  Good start, particularly the first paragraph.  My suggestion is that the cases during COVID-19 are different to those in normal period. So, exclude discussing COVID-19 as your study was conducted in 2024, unless you specifically want to discuss a section on leprosy care management during the pandemic. Similarly, avoid having references relevant to the COVID-19 period accordingly as the scenario would be very different in COVID-19 period.  Discussions are well-detailed out. However, it would be more relevant to discuss your points referencing literature relevant to countries, preferably South Asia,, with similar healthcare and sociocultural systems. Integrate some major constructs, such as age, urban/rural differentials may also play important factor in diagnosis and treatment to leprosy. Also, integrate some important policy measure improvements. | Thank you for the feedback. COVID-19-related discussions are removed from the main manuscript.  Relevant literatures of South Asian countries with similar healthcare and socio-cultural systems wherever relevant are discussed throughout the discussion section in the main manuscript.  Some major constructs, such as age, urban/rural differentials may also play important factor in diagnosis and treatment to leprosy are added in the discussion section of main manuscript.  “ The findings of our study indicate that demographic characteristics, particularly age and remote residence, play a critical role in access to leprosy diagnosis and continuity of treatment. Older adults and people from remote areas experienced delays in leprosy services due to transportation difficulties and a lack of awareness. Similar urban-rural disparities have been confirmed in India and Bangladesh”  Some important policy measure improvements have been recommended in the main manuscript.  “ Policy-level improvements are needed to improve the fidelity of leprosy care delivery in health post of Rupandehi district. Prioritized interventions, such as investing in skin smear services at the local health post, are critical to ensuring swift and accurate diagnosis. Integrating complication management into leprosy training is critical for equipping healthcare providers with complication management skills. Furthermore, strengthening physical infrastructure at health posts allows for patient confidentiality and comfort, which are crucial for reducing stigma as well as increasing treatment adherence.” | #Page 32-39  #Page 37  Lines 659-663  #Page no 38 and 39  Lines 684-690 |  |
| **References**  Please refer to the author's guidelines to format your references appropriately.   Please be very specific when you have headings, subheadings in your study. Follow author guidelines to correctly represent the cases and levels of headings/sub-headings. | References have been updated as per the author's guidelines.  Cases and the level of heading issues have been fixed throughout the manuscript. |  |  |
| **Overall**  Leprosy has traditionally been an important public health concern in Nepal, particularly in rural context. This study needs a revised title; otherwise, the study represents an important work, reflecting the gaps in diagnosing and treating leprosy in Nepal. Methodology is appropriate with different methods to cross-validate the information obtained. The introduction can be framed better with suggested improvements, while results and discussion sections seem too lengthy and need tightening. | Thank you so much for the feedback. Modifications are made in the main manuscript as per suggestion.  The introduction is framed as per feedback, whereas results and discussion are revised as per the suggestion throughout the manuscript. |  |  |
| **Reviewer #2** |  |  |  |
| Very well written paper. No obvious comments to add. Just to make clarification in the manuscript- the basic procedures like VMT/ST and skin smear tests are not performed in government health facilities in Nepal. This makes diagnosis of leprosy delayed. This can be accepted for publication. | Thank you for the comment. VMT/ST is performed every 3 month. Skin smear test are performed in government hospital but health post lack trained health personnel and diagnostic test to perform skin smear test. |  |  |
