# Supplementary material for: Implementation fidelity in leprosy care and support for disability prevention and management in Rupandehi, Nepal: A qualitative study
Source: PLoS One. 2025 Jul 9;20(7):e0327465. doi: 10.1371/journal.pone.0327465 (PMC12240290; doi:10.1371/journal.pone.0327465)
Supplement: Attachment — Submitted filename: Author_Response_Leprosy_Implementation_Fidelity.docx_auresp_2.docx [file pone.0327465.s006.docx]

Editor-in-Chief
PLOS ONE

**Subject: Revisions for Manuscript [PONE-D-25-09075] – "Implementation fidelity in leprosy care and support for disability prevention and management in Rupandehi, Nepal: A qualitative study"**

Dear Editor,

We sincerely appreciate the time and effort you and the reviewers have invested in evaluating our manuscript. This detailed feedback has been invaluable in helping us to refine and strengthen our work. We have carefully considered all comments and revised our manuscript accordingly. Below, we provide a detailed response to each point raised by the reviewers. All changes have been made in the revised manuscript for easy reference.

Below, we provide a point-by-point response to each comment. We have also uploaded a tracked-changes version and a clean version of the revised manuscript for your review.

We sincerely appreciate your guidance throughout this process. Please feel free to reach out if you have any additional questions or concerns.

On behalf of the co-authors,

Sudip Nepal

[nepalsudip25@gmail.com](mailto:nepalsudip25@gmail.com)

| **Editor’s/Reviewer’s comments** | **Response** | **Corrected page#, and lines#** |
| --- | --- | --- |
| **Additional editors comment** |  |  |
| Dear Author, thank you very much for your revision. Both reviewers have recommended acceptance, but I still have some issues with the paper. Kindly address this so we can move soon to acceptance and publication. | Thank you so much for the feedback. We have addressed your comment to move ahead with the publication process. |  |
| 1. Tables 1 and 2, which are respondent profiles, can be transferred to the supplementary appendix. A brief description of who they are, referencing the supplementary appendix, would be sufficient.   **Additional comments after 2^nd^ revision submission:**  “ 1. Please include your Tables 1 and 2 as part of your main manuscript and remove the individual files. Please note that supplementary tables (should remain/ be uploaded) as separate "Supporting Information" files. | Thank you so much for the feedback. Initially, we had removed Tables 1 and 2, which were respondent profiles from the main document during 2^nd^ revision submission and transferred them to the supplementary appendix 1 but after the 2^nd^ revision submission to journal we got additional request to include Table 1 and 2 as part of the main manuscript so Table 1 and Table 2 has been included again in the main manuscript.  “Thank you for the feedback. Table 1 and Table 2 have been included in the main manuscript, and the individual file has been revised” | #Pages 10-15  From lines 218-233 |
| 2. The reflexivity statement should be rewritten and improved. Reflexivity statement is not just about reflection or critical assessment of biases, etc. It is more about how your backgrounds, beliefs and experiences played a part in the interpretation and development of themes and subthemes. There are many good reflexivity statements from previously published papers; please pattern your statement from it. I want to know what your backgrounds and experiences are and how these have affected the analyses and the generation of themes. | Thank you so much for the constructive feedback. A reflexivity statement has been updated as per the feedback.  “The lead investigator (SN) is a public health professional with over six years of work experience in neglected tropical diseases, including leprosy, at the current study site. SN has completed a Master’s in Public Health (MPH) with a specialisation in Implementation Research (IR). Having witnessed firsthand the barriers to leprosy services, SN approached the research process with extensive knowledge of both policy and ground realities. The data collected in the study were generated in the form of codes and guided by Braun and Clarke’s thematic analysis framework, which influenced the development of themes, including barriers to leprosy care and support and healthcare providers’ adherence to leprosy services.  AP and RAA are a professor and a senior epidemiology and implementation research expert, respectively, with over a decade of expertise in qualitative research. Their specialisation in infectious disease control and health service research contributed significantly to ensuring scientific and methodological rigour. Similarly, AT holds a double degree in MSc. in Public Health and M.A. in Gender Studies; AS is currently pursuing a Master's in Public Health and PCJ completed a Master's in Public Health (MPH). SN, AT, AS and PCJ are familiar with Nepali language and culture and have strong experience in leading qualitative studies on the Nepalese context on a range of public health issues.  All co-authors reviewed the data collection tool and supported the analysis and interpretation of the data. Furthermore, collectively the co-authors agreed on the finalisation of a tool, development of codebook, development of sub-themes and themes, preparation, revision and submission of the manuscript. Throughout the study, all authors approached the study with cultural sensitivity, recognising that their positionality could influence how data is interpreted and analysed.”  This information is updated in the methodology section of the manuscript accordingly. | # pages 17 and 18  From lines 282-304 |
| 3. I want Supplementary Tables 1 and 2 to be put into the main paper and replace Table 3 as it stands now. I want the comparisons to the guidelines be more explicit in the presentation of Results, similar to the Acta Medica Philippina paper I sent to you in the previous round. You are talking about implementation fidelity so what is done should be compared to what is ideal or in the guidelines. I am fine with the framework used; it just needs to be more harmonized and streamlined. Like, for theme 1 healthcare provider's adherence to leprosy services. There should be a comparison to what is in the guidelines, then how reality is different or similar to it. As it stands, you are only presenting what is currently being done, which does not tell us anything about implementation fidelity. | Thank you so much for the feedback.  We have now updated the main manuscript with Table 3, which compares the national leprosy operational guideline and actual implementation of leprosy services similar to the Acta Medica Philippina paper.  The codebook, which was previously Table 3 in the main manuscript, has been moved to the Supplementary Appendix 1 | #Page 19-20  Lines 320-322 |
